# Seizures at the onset of aneurysmal SAH: epiphenomenon or valuable predictor?

**DOI:** 10.1007/s00415-020-10173-2

**Published:** 2020-08-27

**Authors:** Marvin Darkwah Oppong, Marcela Jara Bastias, Daniela Pierscianek, Leonie Droste, Thiemo F. Dinger, Yahya Ahmadipour, Laurèl Rauschenbach, Carlos Quesada, Mehdi Chihi, Philipp Dammann, Michael Forsting, Karsten H. Wrede, Ulrich Sure, Ramazan Jabbarli

**Affiliations:** 1 Department of Neurosurgery and Spine Surgery, University Hospital Essen, University of Duisburg-Essen, 45147 Essen, Germany; 2 Clinic for Neurology, University Hospital, Essen University of Duisburg-Essen, Essen, Germany; 3 Institute for Diagnostic and Interventional Radiology, University Hospital Essen, University of Duisburg-Essen, Essen, Germany

**Keywords:** Aneurysm, Seizures, Subarachnoid hemorrhage, Complications

## Abstract

**Objective:**

Seizures at the onset (SAO) of aneurysmal subarachnoid hemorrhage (aSAH) occur in up to one of every five cases. To date, there is no consensus on causal background and clinical value of these early bleeding-related seizures. This study aimed to analyze the predictors and the impact of SAO in aSAH.

**Methods:**

All aSAH patients from the institutional observational cohort (01/2003–06/2016) were retrospectively reviewed. Patients’ charts and emergency protocols from first responders were screened for the occurrence of seizures in the first 24 h after aSAH. Patients’ baseline characteristics and occurrence of post-hemorrhagic complications were analyzed. Outcome endpoints included in-hospital mortality and poor outcome at 6-month follow-up (modified Rankin Scale > 3).

**Results:**

Of 984 patients included in the final analysis, SAO occurred in 93 cases (9.5%) and were independently associated with younger age (< 51 years, *p* < 0.001), WFNS grade ≥ 4 (*p* < 0.001), aneurysm characteristics (location at the proximal branch of the anterior cerebral artery [*p* = 0.037] and irregular sac [*p* = 0.019]) and admission body temperature > 38.3 ℃ (*p* = 0.008). There was an association between SAO and early complications (early infarcts [*p* = 0.004] and primary decompressive craniectomy [*p* = 0.024]). Only in the subgroup analysis restricted to the younger individuals, SAO independently predicted poor outcome of aSAH (*p* = 0.002).

**Significance:**

Onset seizures following aSAH are rare and most likely related to the severity of early brain injury. Particularly, younger individuals are not only at higher risk for SAO, but are also prone to poor outcome in case of aSAH accompanied with SAO.

**Trial registration number:**

German clinical trial registry (DRKS, unique identifier: DRKS00008749, 06/09/2015)

## Introduction

Alongside with typical clinical symptoms like headache, impairment of consciousness or focal neurological deficits, rupture of intracranial aneurysms might also cause seizures at the onset (SAO). The reported incidence ranges from 6.3 to 19% [1–6].

Previous studies identified younger age [1, 4], clinical [5] and radiographic [4] severity of aneurysmal subarachnoid hemorrhage (aSAH) as well as aneurysm location in the posterior circulation [1] as risk factors of SAO. So far, the true pathophysiologic background and clinical consequences of these seizures at the beginning of aSAH remain unclear. There are conflicting reports on the putative impact of SAO on the outcome of aSAH [1–3, 5]. In particular, better outcome after SAO was reported in the sub-cohort of poor grade aSAH patients suggesting that this event might lead to an over-grading of the initial clinical status due to seizure-associated consciousness alterations [5]. Nevertheless, the impact of early seizures on the clinical course and complications of aSAH, e.g. early brain injury (EBI), delayed ischemic neurological deficit (DIND) or delayed cerebral ischemia (DCI), is not fully understood yet. This study aimed to analyze the incidence, predictors, and the impact of SAO in a large single center observational aSAH cohort.

## Methods

All aSAH patients admitted to our institution between January 2003 and June 2016 were eligible for this study. Patients were excluded if they had a known history of epilepsy and/or preictus intake of antiepileptic drugs.

The study was approved by the Institutional Review Board (Ethik-Kommission, Medizinische Fakultät der Universität Duisburg-Essen, Registration number: 15-6331-BO), and registered in the German clinical trial registry (DRKS, Unique identifier: DRKS00008749).

### Treatment for aSAH

All patients with aSAH commonly underwent angiographic confirmation of the bleeding source by digital subtraction angiography (DSA). Decision on treatment was made in favor of microsurgical clipping or endovascular coiling after interdisciplinary discussion between neuroradiologist and neurosurgeon on call. Patients with clinical and radiographic signs of acute hydrocephalus were treated by insertion of an external ventricular drainage. Chronic hydrocephalus was treated by ventriculoperitoneal shunt insertion. Nimodipine was orally administered for 21 days after ictus. To identify cerebral vasospasm, transcranial Doppler ultrasound was performed daily for a minimum of 14 days after ictus. In case of severe refractory cerebral vasospasm, angiographic intra-arterial application of Nimodipine was performed. In cases of persistent elevation of intracranial pressure (ICP) > 20 mmHg, conservative treatment was initiated. In turn, decompressive craniectomy was performed in case of sustaining ICP refractory to conservative treatment. Decompressive craniectomy on the day of aneurysm treatment was referred to as primary, whereat the remaining cases in the later course were referred to as secondary. Additional routine computed tomography (CT) scan(s) was/were carried out in the first 24 h after treatment and after any further surgical intervention or in case of any clinical deterioration.

### Definition, documentation, and treatment of SAO

Based on the electronic patients’ charts and reports from referring hospitals or emergency staff (first responder), all seizures that occurred during the first 24 h after the onset of aSAH were considered as SAO. We defined seizures as focal or generalized repetitive rhythmic jerking, with or without preceding tonic spasms and with or without loss of consciousness (as persuading described by the patients, a nonmedical witness, emergency staff, or physicians). No antiepileptic treatment was initiated in SAH patients with SAO.

### Data management

The electronic patients’ charts were reviewed for demographic, clinical and laboratory parameters. We used the World Federation of Neurosurgical Societies (WFNS) Score [7] to assess the initial clinical condition and dichotomized the score for further analyses into good (WFNS 1–3) and poor (WFNS 4–5) grades. Radiographic imaging was screened for severity of aSAH using the original Fisher scale [8]. For statistical analysis, aSAH severity was dichotomized into low (Fisher 1–2) and high (Fisher 3–4) grades. Occurrence of intracerebral hemorrhage (ICH) and intraventricular hemorrhage (IVH) was documented, including the severity of ICH (volume [in mL] according to the ABC/2-formula [9]) and IVH (according to the original Graeb score [10]). All new hypodensities that were seen in the follow-up CT scans, not related to surgical approach or ICH, were regarded as cerebral infarcts. All new infarcts documented within 72 h after the bleeding event were defined as early infarcts. In turn, cerebral infarcts occurring in the later course of aSAH were defined as DCI [11]. Aneurysm location was stratified into middle cerebral artery (MCA), internal carotid artery (ICA), proximal anterior cerebral artery (pACA, A1 segment including anterior communicating artery), distal (d) ACA, and posterior circulation (posterior cerebral artery, vertebral artery, and basilar artery). Aneurysm sack size was measured as seen in DSA and morphology was defined as irregular in case of additional lobes or daughter aneurysm as observed in DSA.

Information regarding comorbidities (arterial hypertension, smoking, obesity, hyperlipidemia, diabetes, and thyroid diseases), previous medication (oral antihypertensive medication, acetylsalicylic acid (ASA) and phenprocoumon), drug (opioids, amphetamines, and cocaine), and alcohol abuse was taken from the original patients’ charts and standardized admission protocols. Certain vital signs, laboratory and neurocritical parameters, as well as complications at admission (the maximal and minimal systolic blood pressure [in mmHg], presence of fever [maximal temperature at admission > 38.3 ℃]) and during the whole hospital stay (ICP elevation > 20 mmHg requiring conservative/surgical treatment, total time of mechanical ventilation [in days], occurrence of aneurysm rebleeding and DIND) were extracted from the daily intensive care charts. DIND was defined as clinical deterioration of more than 2 points on the Glasgow coma scale or new neurological deficit without any other explanation [11]. The following laboratory values at admission were also recorded for further analysis: increased C-reactive protein (CRP, > 0.5 mg/dL), leukocytosis (white blood cell count [WBC] > 10/nL), hypo- and hypernatremia (serum sodium level < 135 and > 145 mmol/L, respectively), hypo- and hypercalcaemia (serum calcium level < 2.1 and > 2.7 mmol/L, respectively). Finally, in-hospital mortality and poor outcome, defined as a modified Rankin scale (mRS) score > 3 at 6-month follow-up, were used as outcome endpoints.

### Statistical analysis

We used SPSS 22 for Windows (IBM Corp.) and PRISM v. 5.0 (GraphPad Software) for all statistical analyses. Significance level was set to *p* < 0.05. Univariate analyses were performed for all parameters to check for predictors regarding occurrence of SAO and their influence on clinical course and outcome following aSAH. Chi-square test was used for dichotomized variables and for samples with a size smaller than 5 Fisher’s exact test was used. Continuous variables were tested with the Student’s t test for normal distributed data and with the Mann–Whitney *U* test for non-normal distributed data. Significant parameters were included into multivariate regression analysis to identify independent predictors. Multivariate analysis was performed in two steps. First, parameters that were present before, those characterizing SAH event and laboratory parameters at admission were analyzed separately. Then, parameters identified in both analyses were included to the final multivariate model. For continuous variables, common cut-offs (as mentioned above) or those based on the receiver operating curve analysis were applied prior to inclusion to the multivariate analysis. Missing data were managed utilizing multiple imputations.

## Results

**Fig. 1:**
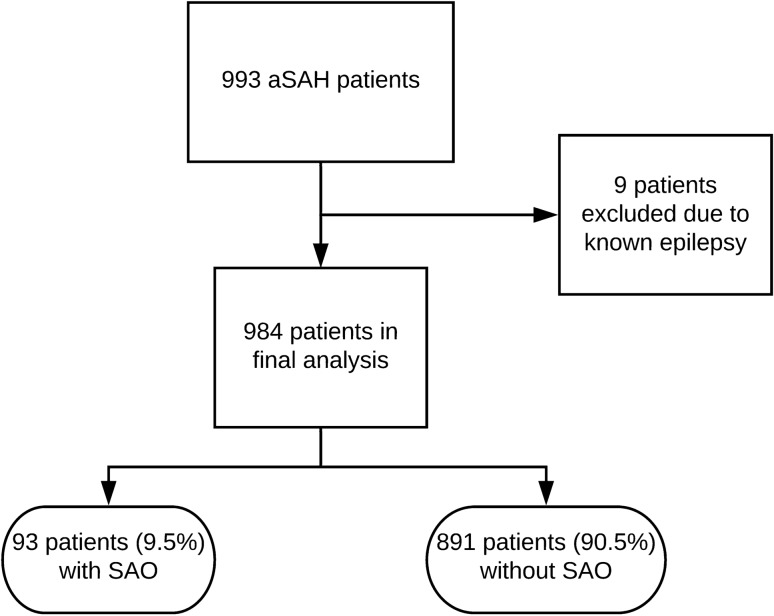
Overview of the whole aSAH cohort, inclusion into final analysis and occurrence of SAO

A total of 984 patients were included in the final analysis. SAO were documented in 93 patients (9.5%, see the flowchart in the Fig. 1). The mean age of the cohort was 55 years ± 14 years standard deviation (SD). Patients were predominately females (67%) and presented with high radiographic severity in 87.4% of the cases. Initial condition was poor in 41.7% of the cases, according to WFNS grading. At 6-month follow-up, 12 patients (1.2%) were lost to follow-up including 1 case (1.1%) with SAO. Baseline characteristics of the cohort are provided in Table 1.

**Table 1:** Overview of baseline characteristics of aneurysmal subarachnoid hemorrhage (aSAH) patients included in the final analysis (*n* = 984)

Parameter	*n*/mean	%/SD
Age (years)	55	± 14
Sex female	659	67.0%
WFNS (4–5)	410	41.7%
Fisher (3–4)*	792	87.4%
ICH	296	30.1%
IVH^†^	445	45.6%
Acute hydrocephalus	699	71.0%
IA location		
MCA	219	22.3%
ICA	116	11.8%
pACA	340	34.6%
dACA	32	3.3%
Posterior circulation	277	28.2%
Treatment (clipping)^‡^	361	38.6%
pDC	180	18.3%
sDC	85	8.5%
Poor outcome (6 months post aSAH)^§^	466	47.9%
In-hospital mortality	179	18.2%

*dACA* distal anterior cerebral artery, *IA* intracranial aneurysm, *ICA* internal carotic artery, *ICH* intracerebral hemorrhage, *IVH* intraventricular hemorrhage, *MCA* middle cerebral artery, *pACA* proximal anterior cerebral artery, *p/sDC* primary/secondary decompressive craniectomy

*Data missing for 78 patients

^†^Data missing for nine patients

^‡^48 patients received no treatment

^§^Data missing for 12 patients

### Demographics and previous medical history and SAO

Patients with SAO were younger compared to seizure-free individuals (48 ± 13 years vs. 55 ± 14 years; *p* < 0.001). Patients’ sex was equally distributed between the two groups (*p* = 0.145). Among premorbid conditions, only drug abuse (5.5% vs. 1.0%; *p* = 0.007; odds ratio (OR) = 5.57; 95% confidence interval (95% CI) 1.83–16.99) showed a predictive value for the occurrence of SAO. Smoking (*p* = 0.576), alcohol abuse (*p* = 0.382), arterial hypertension (*p* = 0.945), obesity (*p* = 0.807), diabetes (*p* = 0.810), thyroid diseases (*p* = 0.322), and hyperlipidemia (*p* = 0.163) did not. A association between preictus medication and SAO was not observed (see Table 2).

**Table 2:** Univariate analysis of influence of seizures at onset (SAO) on treatment and complications following aneurysmal subarachnoid hemorrhage (aSAH)

Parameter	*p*	OR	95% CI
Parameters prior to SAH event			
Demographics			
Age (years)	** < 0.001**	–	–
Sex (female)	0.145	0.72	0.47–1.12
Aneurysm characteristics			
Presence of multiple IA	0.908	0.97	0.62–1.53
IA location (pACA)	**0.042**	1.56	1.01–2.41
IA sac size (mm)	0.147	–	–
Sac irregularity	**0.004**	1.92	1.22–3.02
Premorbid conditions			
Arterial hypertension	0.945	1.02	0.64–1.61
Diabetes mellitus	0.810	0.79	0.28–2.23
Obesity	0.807	1.11	0.49–2.50
Hyperlipidemia	0.163	0.49	0.17–1.37
Thyroid diseases	0.322	0.67	0.30–1.49
Smoker	0.576	1.14	0.72–1.80
Alcohol abuse	0.382	1.39	0.67–2.89
Drug abuse	**0.007**	5.57	1.83–16.99
Medication			
ASA	0.206	0.48	0.15–1.55
Phenprocoumon	0.385	0.31	0.02–5.14
Betablocker	0.879	1.02	0.56–1.86
Calcium antagonist	0.573	1.21	0.60–2.42
ACE inhibitor	0.265	0.70	0.37–1.32
AT1 antagonist	0.687	1.20	0.50–2.88
Statins	0.220	0.37	0.09–1.56
Inital parameters at SAH event			
Clinical and radiographic characteristics			
WFNS grade (4–5)	** < 0.001**	2.67	1.71–4.16
Fisher grade (3–4)	**0.013**	3.39	1.22–9.43
IVH presence	** < 0.001**	2.47	1.58–3.87
IVH severity (oGS)	0.908		
ICH presence	**0.032**	1.61	1.04–2.50
ICH volume (cm^3^)	0.323		
Acute hydrocephalus	**0.002**	2.47	1.37–4.43
Vital parameters at admission			
Maximal systolic blood pressure (mmHg)	0.220	–	–
Minimal systolic blood pressure (mmHg)	0.873	–	–
ICP > 20 mmHg at admission	**0.044**	1.55	1.01–2.38
Fever at admission	** < 0.001**	3.43	1.85–6.33
Laboratory parameters at admission			
Leukocytosis	**0.031**	1.84	1.05–3.22
CRP > 0.5 mg/dl	0.399	0.82	0.52–1.30
Hypernatremia	0.786	1.06	0.36–3.08
Hyponatremia	> 0.99	0.75	0.17–3.25
Hypercalcemia	> 0.99	2.84	0.11–70.47
Hypocalcemia	**0.019**	2.09	1.12–3.91
Treatment			
Treatment modality (clipping)	0.540	1.15	0.74–1.79
No treatment	> 0.99	0.87	0.30–2.46
pDC	**0.024**	1.75	1.07–2.85
sDC	0.422	1.33	0.66–2.67
Clinical course and complications			
IA rebleeding before therapy	**0.003**	2.73	1.39–5.36
ICP > 20 mmHg during hospitalization	0.203	1.39	0.84–2.31
Early infarct	**0.004**	1.86	1.21–2.86
DCI	0.328	1.28	0.79–2.11
DIND	0.221	1.36	0.83–2.22
Chronic hydrocephalus	**0.003**	2.00	1.25–3.19
Duration of mechanical ventilation (days)	**0.001**	–	–
Outcome			
Poor outcome	0.131	1.39	0.9–2.15
In-hospital mortality	0.588	0.85	0.48–1.52

*ASA* acetylsalicylic acid, *DCI* delayed cerebral ischemia, *DIND* delayed ischemic neurological deficit, *IA* intracranial aneurysm, *ICH* intracerebral hemorrhage, *ICP* intracranial pressure, *IVH* intraventricular hemorrhage, *pACA* proximal anterior cerebral artery, *p/sDC* primary/secondary decompressive craniectomy

Statistical significant *p*-values are printed in bold

### Aneurysm characteristics and SAO

**Fig. 2:**
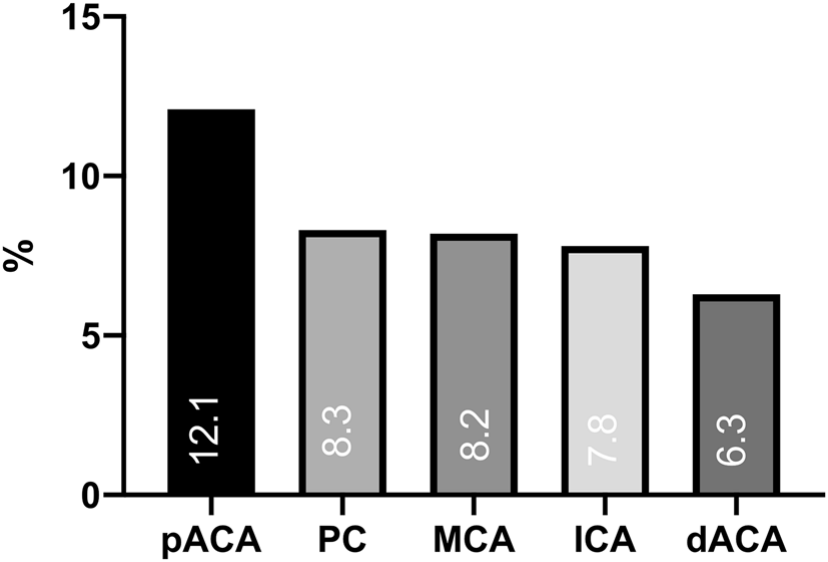
Distribution of SAO risk depending on aneurysm location in percent

Patients with ruptured pACA aneurysm were at higher risk for SAO (12.1% vs. 8.2% for all other aneurysm locations, *p* = 0.042, OR = 1.56, 95% CI 1.01–2.41, Fig. 2). Aneurysm irregularity was also more common among patients with SAO (62.5% vs. 46.4%, *p* = 0.004, OR = 1.92, 95% CI 1.22–3.02). Aneurysm size (*p* = 0.147) and incidence of multiple aneurysm was not different between the two groups (*p* = 0.908) (see Table 2).

### aSAH characteristics and SAO

**Fig. 3:**
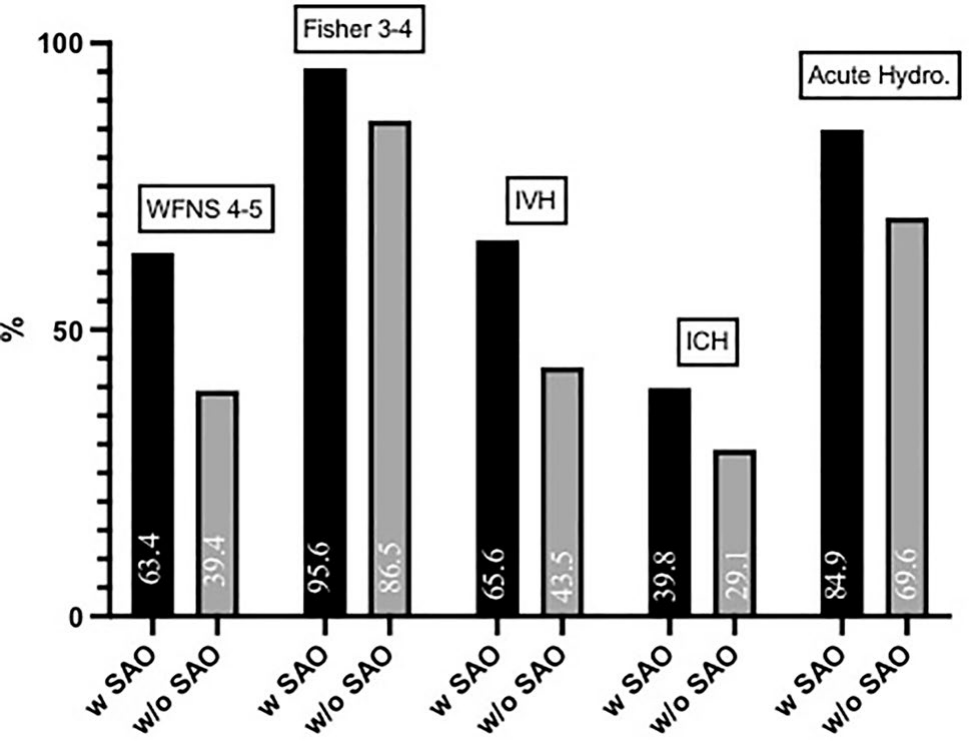
Rate of occurrence of different aSAH characteristics among patients with (w) and without (w/o) SAO

Patients in the SAO group were more likely to present with WFNS = 4–5 (OR = 2.67, 95% CI 1.71–4.16) and Fisher = 3–4 (*p* = 0.013, OR = 3.39, 95% CI 1.22–9.43). The rates of IVH (*p* < 0.001, OR = 2.47, 95% CI 1.58–3.87), acute hydrocephalus (*p* = 0.002, OR = 2.47, 95% CI 1.37–4.43), and of ICH (*p* = 0.032, OR = 1.61, 1.04–2.50) were increased among the SAO group (see Fig. 3). Severity of IVH and volume of ICH did not differ between the two groups (*p* = 0.908 and *p* = 0.323, respectively).

### Further clinical and laboratory parameters at admission and SAO

In the SAO group, incidence of early elevated ICP was higher (50.5% vs. 39.7%; *p* = 0.044, OR = 1.55, 95% CI 1.01–2.38). Moreover, there were higher rates of fever (19.5% vs. 6.6%; *p* < 0.001, OR = 3.43, 95% CI 1.85–6.33), leukocytosis (82.4% vs. 71.8%; *p* = 0.031, OR = 1.84, 95% CI 1.05–3.22) and hypocalcaemia at admission (22.7% vs. 12.3%; *p* = 0.019, OR = 2.09, 95% CI 1.12–3.91) compared with seizure-free patients. Systolic blood pressure at admission and other laboratory parameters showed no differences (see Table 2).

### Treatment and early complications

**Fig. 4:**
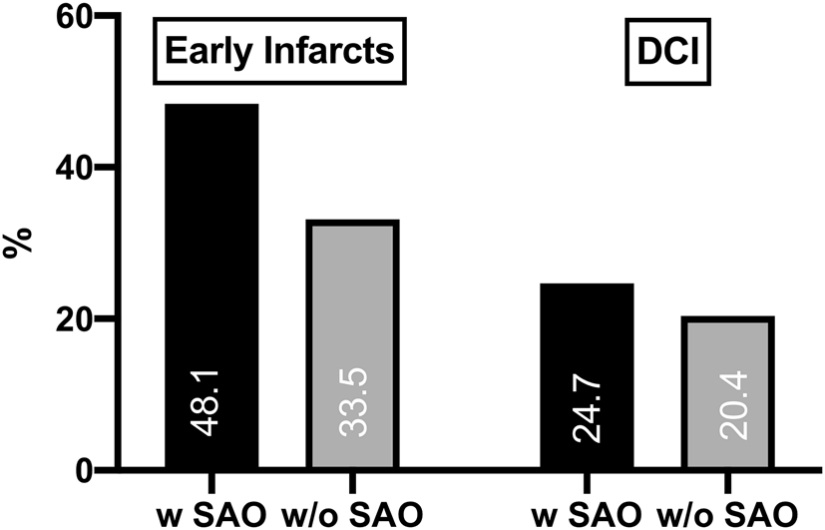
Rate of patients with early infarcts and DCI depending for patients with (w) and without (w/o) SAO

There was no difference regarding the treatment modality (*p* = 0.540) and SAO. At the same time, SAO patients underwent primary decompressive craniectomy more frequently (26.9% vs. 17.4%; *p* = 0.024, OR = 1.75, 95% CI 1.07–2.85). Notably, occurrence of SAO was associated with increased risk for aneurysm rebleeding before therapy (13.0% vs. 5.2%; *p* = 0.003, OR = 2.73, 95% CI 1.39–5.36) and for early infarcts (48.4% vs. 33.5%; *p* = 0.004, OR = 1.86, 95% CI 1.21–2.86, see also Table 2 and Fig. 4).

### Late complications

DCI infarcts (*p* = 0.328, Fig. 4) and DIND events (*p* = 0.221) were not more common in SAO cases. There was an increased hazard for secondary ICP increase (64.1% vs. 42.9; *p* < 0.001, OR = 2.38, 95% CI 1.53–3.73) in SAO patients, but not for the need for secondary decompressive craniectomy (*p* = 0.422). Finally, the patients with SAO required longer mechanical ventilation (8.5 ± 5.9 days vs. 6.3 ± 6.0 days, *p* = 0.001) and developed more frequently a shunt-dependent chronic hydrocephalus (48.1% vs. 31.7%; *p* = 0.003, OR = 2.00, 95% CI 1.25–3.19, see also Table 2).

### Multivariate analysis of SAO predictors

Significant parameters from univariate analysis were included to the multivariate analysis (see Table 3). According to receiver operating area under the curve metrics, patients’ age was dichotomized using the cutoff < 51 years. The final analysis confirmed younger age (< 51 years, *p* < 0.001, adjusted (a)OR = 3.20, 95% CI 1.99–5.15), aneurysm location in the pACA (*p* = 0.037, aOR = 1.63, 95% CI 1.03–2.59), irregular sac morphology (*p* = 0.019, aOR = 1.75, 95% CI 1.10–2.79), poor initial clinical condition (WFNS = 4–5, *p* < 0.001, aOR = 2.40, 95% CI 1.48–3.89) and fever at admission (*p* = 0.008, aOR = 2.49; 95% CI 1.27–4.89) as independent predictors of SAO.

**Table 3:** Multivariate analysis of predictors of seizures at onset (SAO)

Parameter	Step 1			Step 2		
	*p*	aOR	95% CI	*p*	aOR	95% CI
Parameters prior to SAH event						
Age < 51 years	** < 0.001**	**2.89**	**1.83–4.53**	** < 0.001**	**3.20**	**1.99–5.15**
IA location (pACA)	**0.042**	**1.59**	**1.02–2.48**	**0.037**	**1.63**	**1.03–2.59**
Sac irregularity	**0.013**	**1.79**	**1.13–2.83**	**0.019**	**1.75**	**1.10–2.79**
Drug abuse	0.035	3.42	1.09–10.71			
Initial parameters at SAH event						
WFNS grade (4–5)	**0.034**	**1.74**	**1.04–2.91**	** < 0.001**	**2.40**	**1.48–3.89**
Fisher grade (3–4)	0.623	1.33	0.42–4.27			
IVH presence	0.073	1.62	0.96–2.73			
ICH presence	0.618	1.14	0.69–1.88			
Acute hydrocephalus	0.326	1.41	0.71–2.79			
ICP > 20 mmHg	0.881	1.04	0.65–1.67			
Fever	**0.003**	**2.55**	**1.37–4.73**	**0.008**	**2.49**	**1.27–4.89**
Laboratory parameters						
Leukocytosis	**0.030**	**1.87**	**1.06–3.28**	0.309	1.36	0.75–2.46
Hypocalcemia	**0.029**	**1.95**	**1.07–3.54**	0.114	1.64	0.89–3.05

Significant parameters from the step 1 were included in the step 2. All significant p, adjusted odds ratio (aOR) and confidence interval (CI) values are marked bold

*IA* intracranial aneurysm, *ICH* intracerebral hemorrhage, *ICP* intracranial pressure, *IVH* intraventricular hemorrhage, *pACA* proximal anterior cerebral artery

### SAO and impact on outcome

In the whole cohort, univariate analysis revealed no association between the SAO with poor outcome (*p* = 0.131) and in-hospital mortality (*p* = 0.588). In the subgroup analyses based on the two statistically most relevant SAO predictors (patients’ age and initial clinical condition), the stratification according to the WFNS grading showed no impact on the correlation between SAO and outcome endpoints: *p* = 0.350/0.924 (in the WFNS = 1–3 and 4–5 patients, respectively, hereinafter) for poor outcome and *p* = 0.065/ > 0.99 for in-hospital mortality.

In the age-dependent subgroup analysis, there was no impact of the SAO on poor outcome (*p* = 0.729) and in-hospital mortality (*p* = 0.840) in aSAH individuals aged ≥ 51 years-old. In contrast, SAO increased the risk of poor outcome (*p* = 0.002, OR = 2.38, 95% CI 1.37–4.15) and did not affect in-hospital mortality (*p* = 0.674) in younger aSAH counterparts. Finally, independent impact of SAO (*p* = 0.005, aOR = 2.42, 95% CI 1.31–4.47) on poor outcome regardless occurrence of DCI infarcts (*p* < 0.001, aOR = 7.98, 95% CI 4.41–14.44) and aneurysm rebleeding (*p* = 0.008, aOR = 8.68, 95% CI 1.75–42.92) was confirmed in the multivariate analysis for this aSAH sub-population.

## Discussion

This study aimed to evaluate the incidence of SAO, to identify predictors of SAO and to investigate the impact of SAO on clinical course and outcome following aSAH. The incidence of SAO was 9.5% in our cohort and, therefore, in the range of previous publications [1–6, 12]. SAO were independently predicted by younger age, poor initial clinical condition, and fever at admission and aneurysm characteristics (sac irregularity and the location in the pACA). Notably, SAO revealed no impact on the functional outcome in the overall aSAH cohort, but an independent association with poor outcome at six-month follow-up in younger aSAH individuals (< 51 years-old).

### Predictors of SAO

Younger age has already been reported as risk factor for SAO [4, 12]. Additionally, in one series with young aSAH individuals, the rate of SAO was the highest yet documented (21%) [13]. Our study confirms the high frequency of young aSAH patients suffering from SAO. Particularly, the incidence of SAO in individuals aged < 51 years was almost three times higher as in the older patients. As brain volume decreases with age [14, 15], younger individuals are more prone to ICP increase, which in turn, promtes epileptic activity [16].

The severity of aSAH with regard to initial clinical condition [2, 5] and radiographic presentation [2–4] has also been reported as risk factor for SAO. Our study failed to identify the association between the radiographic parameters and SAO occurrence, but confirmed poor initial clinical condition (according to the WFNS grade) as a strong independent predictor of SAO.

Other potential risk factors for SAO assessed in this study should also be mentioned. In particular, drug abuse is an acknowledged risk factor for seizures regardless of aSAH [17]. In the setting of aSAH, it has been shown that cocaine use increases the risk of seizures during hospitalization [18]. In our study, individuals with drug abuse were more likely to present with SAO, but this association has failed to remain significant in multivariate analysis. Another behavioral risk factor associated with seizure risk [17] is alcohol abuse, but it could not be confirmed as SAO predictor. This finding is in line with previous results [2]. Fever has been studied in the context of aSAH mostly during the hospitalization and has been associated with poor outcome and occurrence of cerebral vasospasm [19–21]. Fever at admission, which was independently associated with SAO in our study, might present an early sign of marked neuroinflammatory response to early brain injury (EBI) after aneurysm rupture [22]. In aSAH and traumatic brain injury, occurrence of fever has been interpreted as the consequence of tissue ischemia [23, 24]. Moreover, fever might reflect the severity of initial tissue damage in traumatic brain injury [25], and specifically be related to damage of the frontal lobe [26]. Accordingly, location of the ruptured aneurysm in the ACA has been connected with a higher risk of frontal lobe damage [27].

Besides milder symptoms, hypocalcaemia might also cause seizures [28]. Hypocalcaemia was more common in patients with SAO, but failed to be an independent predictor. Hyponatremia is not an uncommon phenomenon in the clinical course following aSAH [29, 30]. It is suspected that hyponatremia in aSAH patients is usually related to cerebral salt wasting syndrome or inappropriate diuresis [29, 30]. Hyponatremia might ultimately lead to seizures, but rather in the setting of a rapid change of serum sodium levels [31]. In our cohort, sodium levels at admission were not associated with the risk of SAO.

Moreover, aneurysm characteristics also demonstrated significant independent associations with SAO in the present study. Data regarding the relationship between aneurysm location and seizure risk are rare. For instance, Hänggi and colleagues [32] reported on higher epileptogenicity in unruptured aneurysm anatomically related to the temporomedial region. As to the aneurysm morphology, presence of sac irregularity is considered as a sign of aneurysm instability due to local inflammatory processes in the aneurysm wall [33–35]. Whether the link between sac irregularity and SAO occurrence is also related to neuroinflammation and/or EBI severity, should be addressed in future investigations.

### Impact of SAO on clinical course and outcome of aSAH

There was an association between SAO and occurrence of certain complications after aSAH. So, the individuals presenting with SAO were more prone to sustained ICP increases, mostly in the early phase of aSAH. Accordingly, these patients were at higher risk for aneurysm rebleeding before treatment, early intractable ICP increase necessitating primary decompressive craniectomy and occurrence of early infarcts.

In contrast, SAO did not impact the risk of secondary ischemic complications like DCI or DIND. This observation might suggest a link between SAO and the severity of EBI. At the same time, individuals with SAO also showed certain secondary complications like prolonged ICP increase requiring conservative treatment, longer duration of mechanical ventilation, and development of chronic hydrocephalus necessitating shunt placement. These results highlight the need for continuous ICP monitoring in aSAH individuals with SAO.

So far, the impact of SAO on functional outcome has been controversially discussed. Prior studies elaborated no influence [1, 2], negative influence [3, 4] or even a positive influence (in poor grade patients) of SAO on outcome [5]. Our study revealed no impact of SAO on functional outcome for the overall cohort, as well as in different WFNS grade subgroups. However, the analysis restricted to the younger aSAH individuals showed a significantly increased risk of poor outcome at 6-month follow-up in aSAH patients presenting with SAO.

In summary, SAO is a distinct and clinically relevant early complication of aSAH related to the EBI. Onset seizures predominantly affect younger individuals with ruptured aneurysm and are associated with poorer outcome in this specific aSAH sub-population.

## Limitations

The main limitation of this study is its retrospective design. The completeness and reliability of data are limited by the nature of its retrospective assessment. There is a risk of underestimation of SAO rate, especially in aSAH individuals with unobserved bleeding event. On the other side, there is a risk of misinterpretation of seizures observed by nonmedical personal. However, our analysis is based on the largest-to-date aSAH series addressing the SAO event and utilizes multivariate assessment allowing the adjustment of the tested associations for relevant confounders.

## Conclusions

Younger individuals with poor initial clinical condition, fever at admission, irregular aneurysm, and/or aneurysm located in the pACA are more prone to SAO. Strong associations of SAO with early complications of aSAH might indicate a causal relationship between SAO and EBI. Due to sustained ICP increase and more severe disease course, continuous ICP monitoring seems to be crucial in patients with SAO. In younger individuals aged < 51 years, SAO is independently associated with poor outcome of aSAH.

## Data Availability

The data that support the findings of this study are available from the corresponding author upon reasonable request.
